# Spontaneous bladder diverticulum rupture due to a squamous cell carcinoma of the bladder: a case report

**DOI:** 10.1093/jscr/rjaa454

**Published:** 2020-11-28

**Authors:** Wajih Sahnoun, Sami Ben Rhouma, Aziz Kacem, Khaireddine Mrad Dali, Issam Rekik, Beya Chelly, Yosri Messaoudi, Yassine Ouanes, Ahmed Sellami, Yassine Nouira

**Affiliations:** Department of Urology, La Rabta Hospital, Tunis, Tunisia; Department of Urology, La Rabta Hospital, Tunis, Tunisia; Department of Urology, La Rabta Hospital, Tunis, Tunisia; Department of Urology, La Rabta Hospital, Tunis, Tunisia; Department of Urology, La Rabta Hospital, Tunis, Tunisia; Department of Pathology, La Rabta Hospital, Tunis, Tunisia; Department of Anesthesiology and Reanimation, La Rabta Hospital, Tunis, Tunisia; Department of Urology, La Rabta Hospital, Tunis, Tunisia; Department of Urology, La Rabta Hospital, Tunis, Tunisia; Department of Urology, La Rabta Hospital, Tunis, Tunisia

## Abstract

While bladder rupture is most of the time secondary to external injury such as trauma or iatrogenic events, spontaneous bladder rupture (SBR) is a rare condition which is mostly associated with bladder cancer, neurologic bladder or radiotherapy. We report a case of a 63-year-old patient with an invasive squamous cell bladder carcinoma who presented acute peritonitis caused by a SBR while being prepared for radical surgery. CT-scan helped to confirm diagnosis and emergency cystectomy was performed. SBR should be considered in differential diagnosis of peritonitis. On time diagnosis and adequate surgery could improve its prognosis.

## INTRODUCTION

Spontaneous bladder rupture (SBR) is extremely rare and potentially lethal. Mortality rate following a SBR is about 50% but has declined in recent years thanks to better management of complications. SBR most often occurs on a weakened bladder. Clinical presentation is generally that of a peritonitis. Herein, we present an extremely rare case of a spontaneous bladder diverticulum rupture complicating a squamous cell carcinoma (SCC) of the bladder.

## CASE REPORT

**Figure 1:**
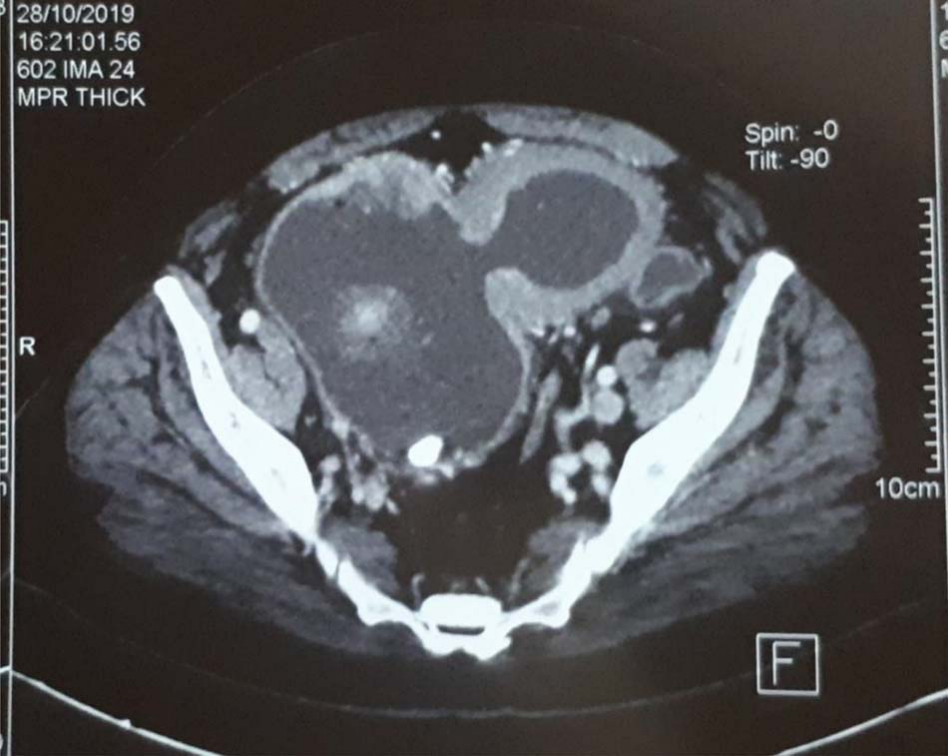
CT-scan of the bladder 2 months before the rupture showing bladder tumor in the dome and in intradiverticular and a bladder stone.

**Figure 2:**
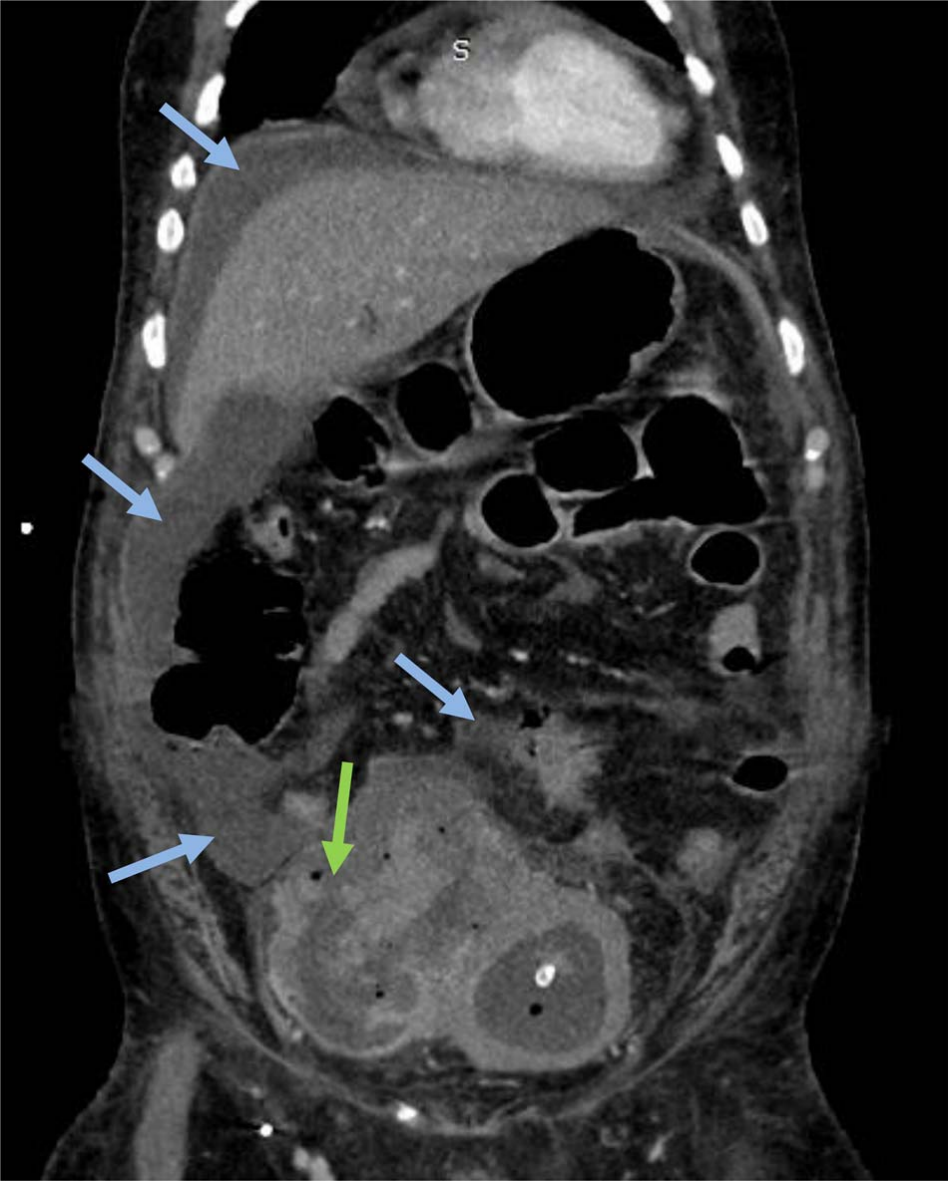
Emergency CT-scan showing intraperitoneal swallowing (blue arrows) due to a fistulization of the bladder (green arrow).

**Figure 3:**
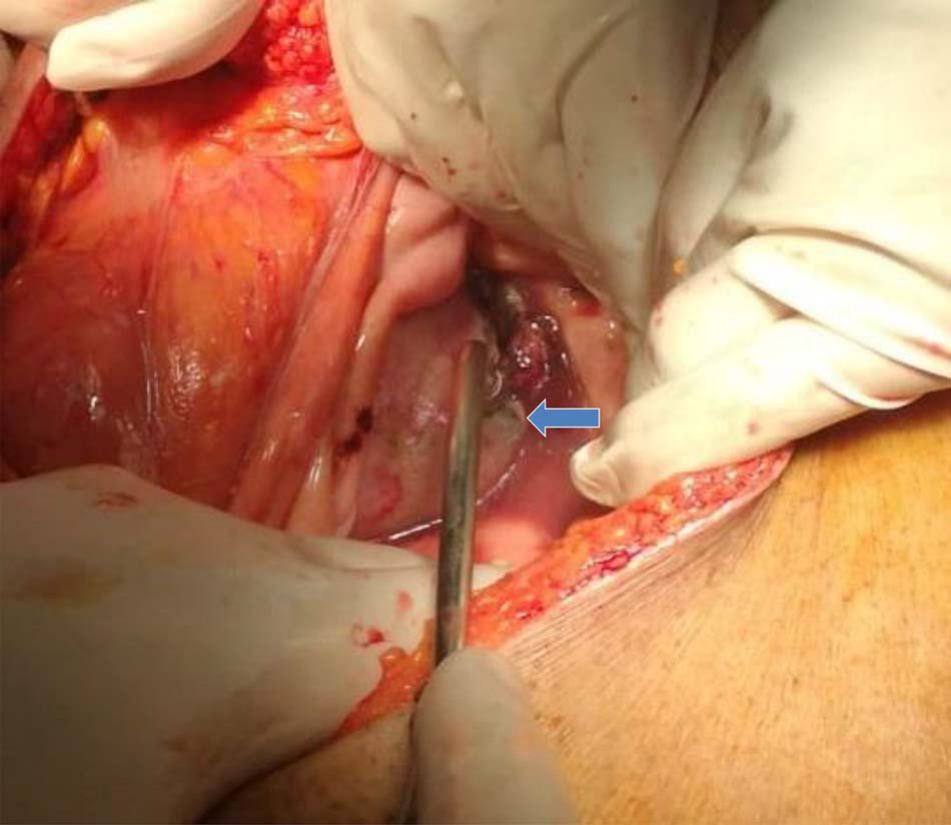
Intraoperative findings included a 2 cm defect at a bladder diverticulum (intraperitoneal view).

A 63-year-old man with a history of transurethral resection of prostate and a ballistic lithotripsy of bladder stone 15 years ago, presented with low urinary tract symptoms and hematuria. Cystoscopy showed multiple bladder stones and a bulky tumor mainly intradiverticular (Fig. 1). Partial resection of the tumor was done and pathology concluded on a squamous cell invasive bladder carcinoma. Since staging showed no metastatic lesion, a cystoprostatectomy was decided. Its execution was delayed because of a pulmonary embolism treated with curative anticoagulation and a severe paraneoplastic hypercalcemia treated with Zoledronic acid and veinous hydration on hospitalization. At Day 10 from admission and Day 60 after endoscopic resection of the tumor, the patient complained of abdominal pain, with diffuse tenderness and fever. Biology shows biologic inflammatory syndrome and kidney failure. Peritonitis was suspected and CT-scan showed a perforated bladder diverticulum with intraperitoneal effusion (Fig. 2). An emergency surgical investigation was executed, objecting a peritoneal cavity filled with nauseating hematic urine derived from a 2 cm disruption at the level of a posterolateral bladder diverticulum (Fig. 3). Radical cystectomy was performed. The patient was in severe septic shock requiring catecholamines and the procedure had to be shortened. No pelvic lymphadenectomy was done and bilateral ureterostomy was chosen as urinary diversion. The intervention lasted 2 h and there was no significant blood loss.

**Figure 4:**
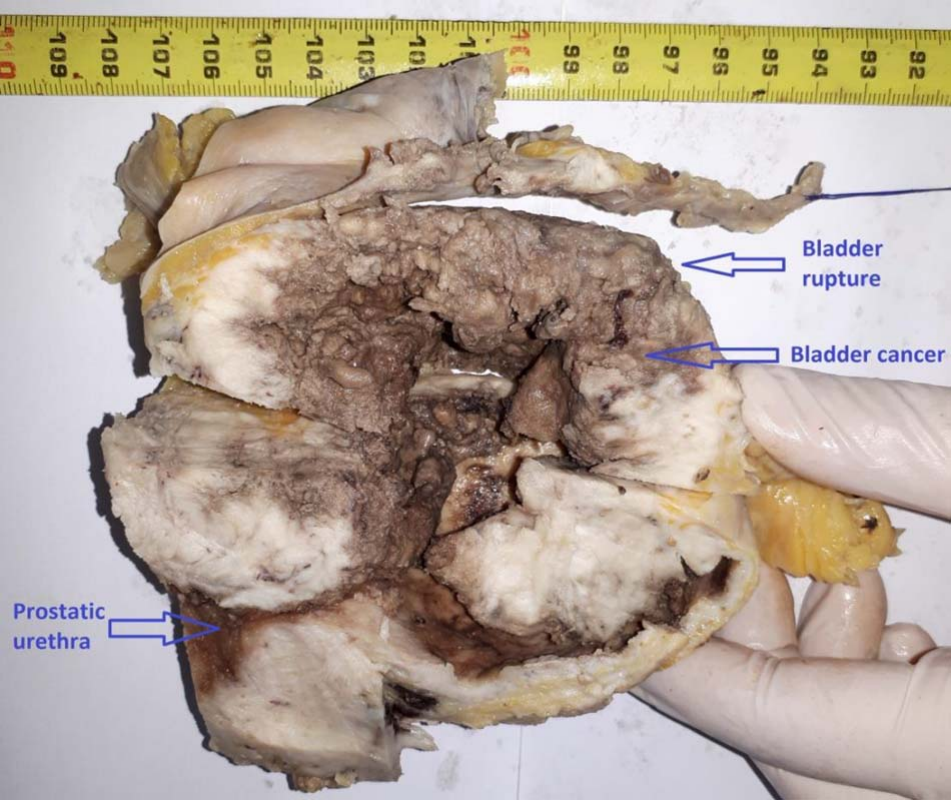
Macroscopic appearance of rupture in a tumor bladder.

**Figure 5:**
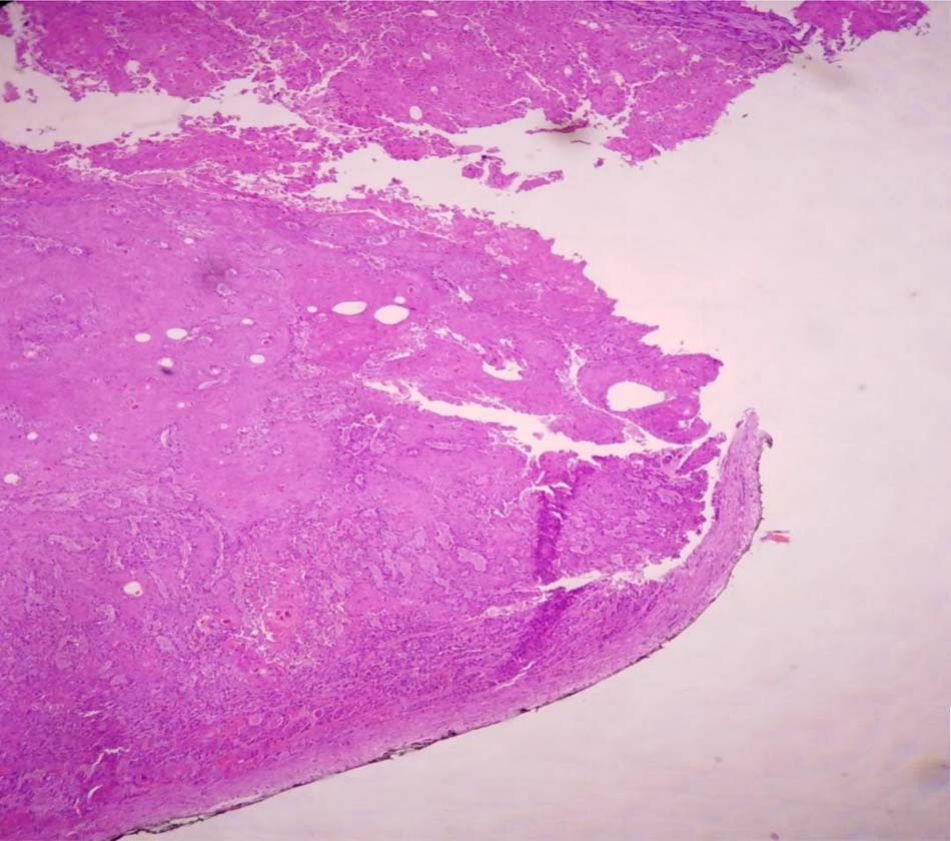
H & E staining viewed under x10 with Olympus CX23 showing bladder wall infiltration with a SCC perforating serosa (bladder surface inked).

**Figure 6:**
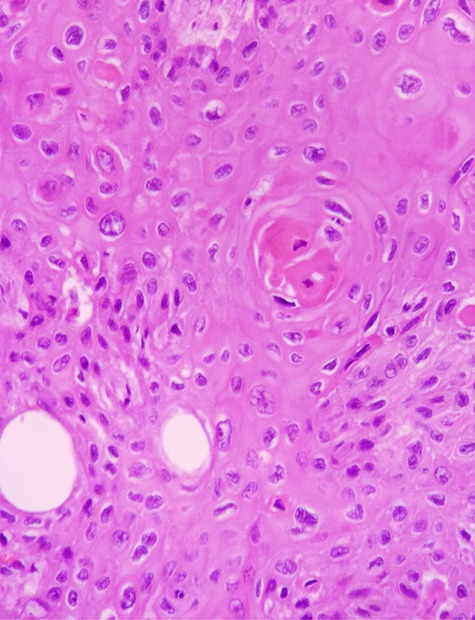
H & E staining viewed under x40 with Olympus CX23 showing a well differentiated SCC (Presence of keratin pearls and intercellular bridges).

Postoperative course was marked by an aggravation of the septic shock, multiorgan failure, and death of the patient at Day 2 after surgery. Pathological examination concluded a pure SCC that massively invades the bladder muscle and perivesical fat (Figs 4–6).

## DISCUSSION

SCC of the bladder represents 2–5% of bladder tumors in most contemporary cystectomy series in western countries [1]. Nonbilharzial SCC is usually associated with chronic irritation of the bladder. This irritation is mainly due to bladder stone, indwelling catheters, recurrent urinary tract infections and exposition to cyclophosphamides [2]. When bladder irritation is caused by bladder stones, it is usually giant stones. Fernando *et al.* [3] reported the case of a patient with SCC of the bladder. This patient has been operated 3 years ago for a bladder stone measuring 5.6 cm. In our case, the patient had about 10 bladder stones of 1–2 cm in diameter each. SCC of the bladder is most often invasive at the time of diagnosis and has significantly higher mortality than urothelial carcinoma. In a large series of 1422 patients treated for non-bilharzian SCC, 85% were pT2 at the time of diagnosis [4]. SBR has a very low incidence (1:126 000). Tumor bladder rupture has even a lower incidence. Mortality in this case is 47% [5]. SBR occurs more frequently in male patients.

SBR is rarely idiopathic. Most often there is a condition weakening the bladder such as urinary tuberculosis, chronic cystitis, indwelling catheter, bladder diverticulum, pelvic radiotherapy, neurologic bladder, bladder tumor and alcohol intoxication [6]. Our patient had a transurethral catheter for 2 months, had SCC of the bladder and bladder diverticuli. As in our case, the clinical presentation is that of acute peritonitis with renal failure caused by peritoneal absorption of urine [7]. Although the incidence of SCC is much lower than that of urothelial carcinoma, both of them are equally present when rupture occurs in bladder tumor. This suggests that SCC is more often complicated by perforation than urothelial carcinoma [8].

The rupture site is most often at the bladder dome, although there have been reported ruptures at the floor or at a lateral face. Sub-peritoneal ruptures generally evolve favorably under bladder drainage, while intraperitoneal ruptures require urgent surgery ranging from cystorraphy to partial or total cystectomy. From a carcinological point of view, radical cystectomy is the ideal treatment for bladder tumor rupture. It should be performed whenever the patient’s condition allows it [9].

Prognosis after surgical treatment of SBR remains poor and the majority of patients die within 8 months according to a literature review by Ahmed *et al.* [8]. An unnoticed spontaneous rupture of the bladder has an even poorer prognosis with a mortality rate of 80% [7]. This underlines the importance of timely diagnosis. In the case of our patient, surgical exploration was in favor of a quite old peritonitis, explaining the refractory septic shock and the resulting death. In fact, peritoneal signs and renal failure were discreet and progressive because the patient was under prophylactic antibiotic before radical gesture and had a urinary catheter for a urine retention and thus the intraperitoneal flow of the urine was slower. Abdominal CT-scan confirmed the suspicion. The contribution of CT-scan to the diagnosis of bladder rupture, especially in doubtful cases, has been well demonstrated by Lowe *et al.* [10].

## CONCLUSION

Although rare, SBR is to suspect in presence of peritoneal signs and a renal failure especially when there are conditions that can weaken the bladder, including bladder carcinoma. Prognosis is related to the time taken to manage the patient but remains quite bad in all cases.
